# Synergistic Antiproliferative Effects of All-Trans Retinoic Acid and Paclitaxel on Autosomal Dominant Polycystic Kidney Disease Epithelial Cells

**DOI:** 10.1155/2021/1242916

**Published:** 2021-10-06

**Authors:** Que Thanh Thanh Nguyen, Thi Xoan Hoang, Hyunjin Ryu, Kook-Hwan Oh, Jae Young Kim

**Affiliations:** ^1^Department of Life Science, Gachon University, Seongnam, Gyeonggi-Do 13120, Republic of Korea; ^2^Department of Internal Medicine, Seoul National University College of Medicine, Seoul, Republic of Korea

## Abstract

Autosomal dominant polycystic kidney disease (ADPKD) is a genetic disorder characterized by uncontrollable epithelial cell growth, cyst formation, and kidney malfunction. In the present study, we investigated the antiproliferative effects of the treatment with the combination of paclitaxel (PAC) and all-trans retinoic acid (ATRA) on ADPKD epithelial cells. Our results show that the combined treatment with 1 nM PAC and 10 nM ATRA significantly suppressed ADPKD cell proliferation (20%), while the treatment with ATRA or PAC alone had no such effect. Treatment with PAC and ATRA induced cell cycle arrest at the G2/M phase and apoptosis by upregulating p53 and caspase-8 expression and increased the intracellular calcium (Ca^2+^) level possibly by enhancing Ca^2+^ uptake via plasma membrane channels. In addition, this treatment suppressed extracellular signal-regulated kinase signaling possibly through mitogen-activated protein kinase phosphatase-1 activation. Thus, the combination of PAC and ATRA can be explored as a potential treatment regimen for ADPKD.

## 1. Introduction

Polycystic kidney disease (PKD) is a life-threatening inherited monogenic disorder characterized by the progressive decline in the kidney function resulting from the renal cysts owing to enhanced cell proliferation [1, 2]. There are two pure forms of PKD, namely, autosomal dominant PKD (ADPKD) and autosomal recessive PKD (ARPKD). ADPKD has a much higher frequency (1 : 400 to 1 : 1,000) than ARPKD [1]. ADPKD can be caused by mutations in either one of the two genes, *PKD1* in 85% cases or *PKD2* in 15% cases [3–5]. The *PKD1* gene encodes polycystin-1 (PC-1) located in the plasma membrane. By interacting with the integral membrane PC-2, which is encoded by *PKD2*, the PC-1/2 complex produces cation-permeable currents that are involved in the modulation of the intracellular calcium (Ca^2+^) uptake or cell division [4]. Dysfunction of PC-1/2 may lead to alternative Ca^2+^ homeostasis to activate cyclic adenosine monophosphate (cAMP) and triggers uncontrollable cell proliferation, which initiates cyst formation in ADPKD [4].

Although there is no successful treatment for ADPKD, an initial study with taxol suggested the central role of microtubule cytoskeleton in the pathogenesis of PKD in a mouse model (cpk mouse) and the potential applicability of microtubule inhibitors structurally similar to taxol for PKD treatment [6]. Paclitaxel (PAC), a mitotic inhibitor, is one of the most effective anticancer drugs. Mechanisms underlying the anticancer effect of PAC involve disruption of the mitotic spindle formation followed by cell cycle arrest at the G2/M phase [7], induction of apoptosis by Ca^2+^ homeostasis modulation [8], activation of proapoptotic signaling [9], upregulation in the expression of the genes encoding p53 and caspases [10], and inhibition of the mitogen-activated protein kinase (MAPK) signaling pathway [11, 12]. However, adverse effects associated with PAC, including thrombocytopenia, leukopenia, anaemia, alopecia, nausea, and vomiting, have raised concerns [13].

All-trans retinoic acid (ATRA) is an active form of vitamin A that regulates various cellular activities, including proliferation, differentiation, apoptosis, and cell cycle arrest [14–16]. In combination with other therapeutic agents, ATRA was reported to increase the efficacy of various anticancer agents [17–20]. In particular, ATRA has been shown to potentiate the antitumor effects of PAC [21]. Therefore, we examined the plausible antiproliferative effects of the combination treatment with PAC and ATRA on ADPKD epithelial cells and elucidated the mechanisms underlying these effects.

## 2. Materials and Methods

### 2.1. Chemicals and Antibodies

PAC, ATRA, BMS753, LG100268, BMS195614, and verapamil were purchased from Sigma-Aldrich (St. Louis, MO, USA). UVI3003, 3,5-bis(trifluoro-methyl)pyrazole (BTP-2), and NSC-95397 and antibodies for phospho-MAPK kinase (p-MEK)-1/2, p-extracellular signal-regulated kinase- (pERK-) 1/2, ERK-1/2, p53, cyclin B1, caspase-3, and *β*-actin were supplied by Santa Cruz Biotechnology (Dallas, TX, USA). Antibodies for caspase-8 and caspase-9 were procured from Cell Signaling Technology (Danvers, MA, USA).

### 2.2. Cell Culture

The ADPKD epithelial cell line (WT 9-7) and the primary normal human renal mixed epithelial cells (HRECs) were purchased from the American Type Culture Collection (ATCC; Manassas, VA, USA) and cultured in Dulbecco's modified Eagle's medium (DMEM; WelGENE Inc., Seoul, South Korea) supplemented with 10% heat-inactivated fetal bovine serum (FBS), 100 *μ*g/mL penicillin, and 100 *μ*g/mL streptomycin (Invitrogen Corp, Carlsbad CA, USA) at 37°C in a 5% CO_2_ humidified incubator.

### 2.3. Cell Proliferation, Apoptosis, and Cell Cycle Assays

For cell proliferation analysis, ADPKD cells and HRECs were seeded onto 96-well culture plates at a density of 1 × 10^4^ cells/well and treated with various concentrations of PAC and ATRA for different time periods. Cell viability was determined using an EZ-Cytox cell viability assay kit (DOGEN, Seoul, South Korea) according to the manufacturer's instructions.

To examine whether the antiproliferative effects of the combination treatment with PAC and ATRA are associated with the retinoid acid receptor (RAR) and retinoid X receptor pathway, cells were treated with 100 nM RAR*α* or RXR*α* agonist together with 1 nM PAC in the absence of ATRA or with 100 nM RAR*α* or RXR*α* antagonist in the presence of 1 nM PAC and 10 nM ATRA.

To assess cell migration, a wound healing assay was performed. ADPKD cells were grown to confluence in six-well plates at a density of 1 × 10^5^ cells/well after 24 h. A wound was created by scratching the cell monolayer in a straight line using a sterile P-200 pipette tip. Next, cells were washed at least thrice with Dulbecco's phosphate-buffered saline (DPBS) to remove nonattached cells or debris and then treated with 1 nM PAC and 10 nM ATRA. The scratch width was recorded at various time points from 0 to 48 h posttreatment. The speed of gap closure was determined from the gap size using Olympus cellSens Standard software.

To determine apoptosis, cells were seeded onto six-well culture plates at a density of 1 × 10^5^ cells/well and incubated with 1 nM PAC and 10 nM ATRA for 48, 72, or 96 h. After treatment, cells were incubated with fluorescein isothiocyanate- (FITC-) Annexin V in a buffer containing propidium iodide (PI) for 15 min at 25°C in the dark. Then, fluorescence signals were analyzed by Cytomics FC500 MLP (Beckman Coulter Inc., Fullerton, CA, USA). The percentage of apoptotic cells was calculated as the total percentage of end-stage apoptotic cells and early-stage apoptotic cells.

To examine cell cycle arrest, cells were seeded onto six-well culture plates at a density of 1 × 10^5^ cells/well. After 48 h of treatment with 1 nM PAC and 10 nM ATRA, the cells were fixed with 4% formaldehyde for 15 min at 25°C. Next, the cells were washed twice with DPBS and stained with PI-containing RNase at 25°C for 30 min in the dark. Finally, cell cycle analysis was performed on Cytomics FC500 MLP.

### 2.4. Fluorescence Microscopy

Cells were cultured on a confocal dish at a density of 1 × 10^5^ cells/dish and then treated with 1 nM PAC and 10 nM ATRA. After 48 h treatment, cells were fixed with 4% formaldehyde for 15 min and stained with Hoechst 33342 for 30 min. The photographs were observed by fluorescence microscopy.

To detect intracellular calcium signal by fluorescence microscopy, cells were seeded on the confocal dish with a density of 1 × 10^5^ cells per dish and treated with 1 nM PAC and 10 nM ATRA. After 72 h treatment, cells were fixed with 4% formaldehyde for 15 min and incubated with a permeabilization buffer at 25°C for 15 min. Cells were mixed with 50 *μ*L Flou-4-NW-dye purchased from Molecular Probes (Invitrogen) and incubated for 30 min at 37°C in the dark. Next, cells were placed at 25°C for 30 min and stained with Hoechst 33342 for 30 min. The cells were observed by fluorescence microscopy.

### 2.5. Flow Cytometry

To examine intracellular Ca^2+^ levels, cells were seeded onto six-well culture plates at a density of 1 × 10^5^ cells/well. After treatment, the cells were collected and washed with calcium-free DPBS (Sigma-Aldrich). The cells were then incubated with 50 *μ*L Flou-4-NW-dye for 30 min at 37°C in the dark, followed by incubation at 25°C for 30 min. The fluorescence was measured by Cytomics FC500 MLP.

To examine intracellular protein expression, cells were seeded onto T25 culture flasks at a density of 2.5 × 10^5^ cells/flask and treated with the combination of 1 nM PAC and 10 nM ATRA for 72 h. Collected cells were suspended in a permeabilization buffer, centrifuged, and stained with primary antibodies for p-MEK-1/2, p-ERK-1/2, or ERK-1/2 diluted in the permeabilization buffer. After 30 min of incubation, cells were centrifuged and washed twice with DPBS. After incubation with an Alexa Fluor 488-conjugated mouse IgG secondary antibody (A-6421, Invitrogen) or Alexa Fluor 488-conjugated rabbit IgG secondary antibody (sc-516248, Santa Cruz Biotechnology) for 30 min at 4°C, fluorescence signals were recorded on Cytomics FC500 MLP.

### 2.6. Western Blot Analysis

ADPKD cells were seeded onto T25 culture flasks at a density of 2.5 × 10^5^ cells/flask. The cells were incubated with test agents depending on the experiments. After treatment, cells were lysed with Triton X-100 lysis buffer containing protease inhibitors. The supernatant from whole-cell lysates was collected by centrifugation. For p53 activation, nuclear and cytoplasmic proteins were separated using the NucBuster Protein Extraction Kit (Novagen, Rockland, USA). Protein concentrations were determined using the Bradford assay. Later, 30 *μ*g total protein was subjected to 10% sodium dodecyl sulfate-polyacrylamide gel electrophoresis, and the separated protein bands were blotted onto polyvinylidene fluoride membranes (Bio-Rad). The membranes were blocked with 5% bovine serum albumin and then probed with primary antibodies for p-MEK-1/2, p-ERK-1/2, ERK-1/2, p53, caspase-9, caspase-8, caspase-3, or *β*-actin. The protein of interest was detected by incubation with secondary horseradish peroxidase-conjugated goat anti-mouse (Santa Cruz Biotechnology) or donkey anti-goat antibodies (Santa Cruz Biotechnology) and visualized with an enhanced chemiluminescence solution from GE Healthy Care Life Science using a ChemiDoc MP system (Bio-Rad). *β*-Actin was used as a control.

### 2.7. Statistical Analysis

The experiments were conducted at least thrice, and all data were independently expressed as the mean ± standard deviation (SD). Significant differences among groups were analyzed by one-way analysis of variance (ANOVA) followed by Tukey's HSD using SPSS 12.0 for Windows. Differences with *p* values < 0.05 were considered significant.

## 3. Results

### 3.1. Combination Treatment with PAC and ATRA Synergistically Inhibits the Proliferation of ADPKD Cells but Not HRECs

**Figure 1:**
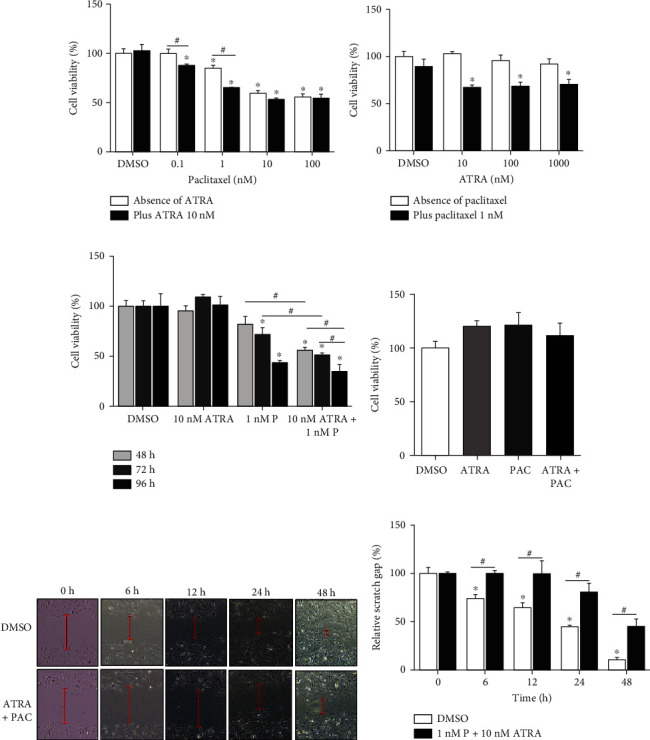
Combination treatment with PAC and ATRA synergistically inhibits ADPKD cell proliferation. WT 9-7 cells were treated with PAC and ATRA. (a) Cells were treated with 10 nM ATRA plus various concentrations of PAC (0.1 to 100 nM) for 48 h. (b) Cells were treated with 1 nM PAC plus various concentrations of ATRA (10 to 1000 nM) for 48 h. (c) Cells were treated with 10 nM ATRA alone, 1 nM PAC alone, or 10 nM ATRA plus 1 nM PAC for different time periods (48, 72, or 96 h). (d) HRECs were treated with 10 nM ATRA plus 1 nM PAC. After treatment, cells were harvested, and their viability was determined by the EZ-Cytox assay. (e) After 24 h of culture, a wound was created by scratching the WT 9-7. Cell monolayer with a micropipette tip (200 *μ*L). The cells were then treated with 1 nM PAC plus 10 nM ATRA. The red dotted lines indicate the wound gap at the beginning of the assay; the wound gap was recorded at 0, 12, 24, and 48 h postscratching. Magnification 40x, scale bar 200 *μ*m. (f) The relative scratch gap was calculated as the ratio of the measured size of the gap at the given time point and the initial gap size at 0 h. Data are expressed as the mean ± SD. ^∗^*p* < 0.05 vs. DMSO, ^#^*p* < 0.05.

To determine the optimal concentrations of PAC and ATRA suitable for the combination treatment, cells were treated together with different concentrations of PAC and ATRA. Treatment with 10 nM PAC alone inhibited cell viability by approximately 40% (Figure 1(a), white bars), while different concentrations (10 to 1000 nM) of ATRA alone had no significant inhibitory effect on cell viability (Figure 1(b), white bars). We fixed ATRA concentration at 10 nM and used different concentrations of PAC for the combination treatment and found that 1 nM PAC showed the most potent inhibitory effect (20%) on cell viability in combination with ATRA as compared with PAC alone. Other PAC concentrations were deemed less effective (12% at 0.1 nM, 6% at 10 nM, and 1% at 100 nM, respectively) (Figure 1(a)). Therefore, we fixed the concentration of PAC at 1 nM and used different concentrations of ATRA and observed the maximum inhibitory effect on cell viability in the presence of at least 10 nM ATRA in combination with PAC (Figure 1(b)). These optimal concentrations of 1 nM for PAC and 10 nM for ATRA were confirmed by subsequent experiments with different incubation periods (Figure 1(c)). Thus, we used the combination of 1 nM PAC and 10 nM ATRA (hereafter PAC+ATRA) in further experiments. Next, we determined whether the combination treatment affects the viability of normal HRECs, as these drugs are expected to selectively work on ADPKD cells without inducing toxicity to normal cells. As shown in Figure 1(d), treatment with PAC+ATRA did not affect HREC viability. To confirm the antiproliferative effect of PAC+ATRA on ADPKD cells, we performed a wound healing assay (Figures 1(e) and 1(f)). Cell migration and wound healing were observed over time in both groups. Wound gap closure was much slower in the PAC+ATRA group than in the dimethyl sulfoxide (DMSO) control group, indicating that the combination treatment with PAC+ATRA suppresses the proliferation and migration of ADPKD cells.

### 3.2. Combination Treatment with PAC and ATRA Induces G2/M Phase Cell Cycle Arrest and Apoptosis by Upregulating p53 and Caspase-8 Expression

**Figure 2:**
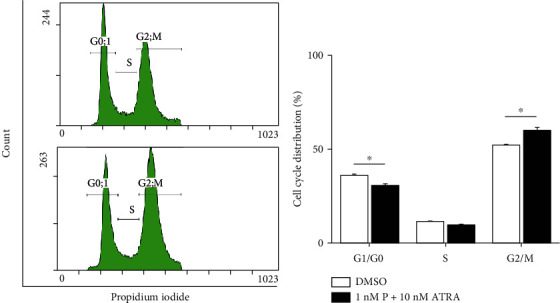
Combination treatment with PAC and ATRA induces cell cycle arrest at the G2/M phase. WT 9-7 cells were treated with DMSO or the combination of 1 nM PAC and 10 nM ATRA for 48 h. To examine cell cycle arrest, the cells were fixed with 4% formaldehyde for 15 min and then incubated with PI in a buffer containing RNase and analyzed by flow cytometry. ^∗^*p* < 0.05.

**Figure 3:**
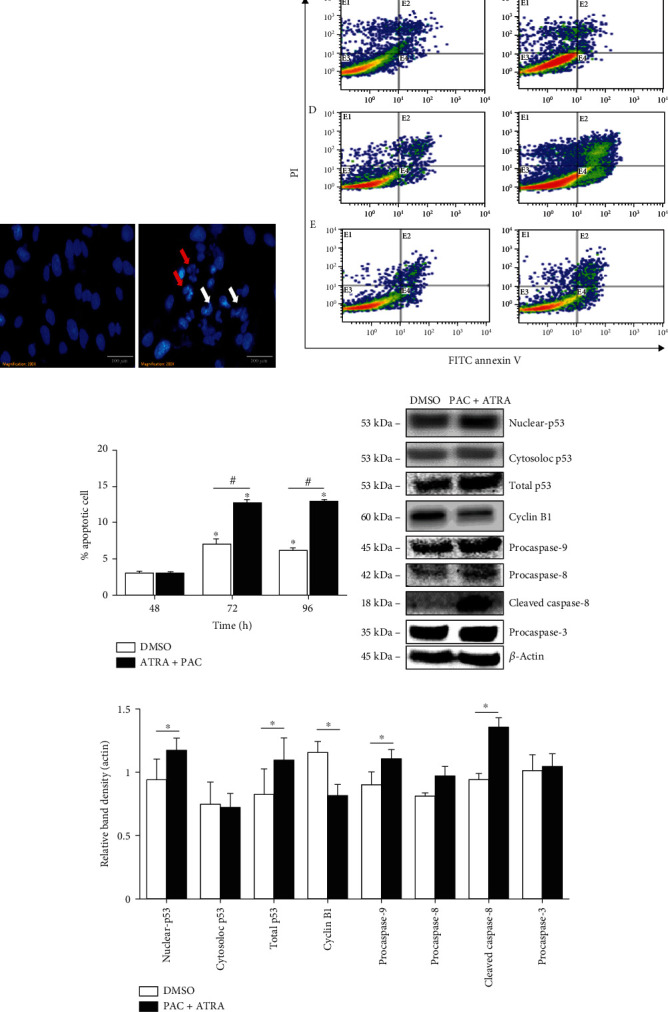
Combination treatment with PAC and ATRA induces apoptosis by upregulating p53 and caspase-8 expression. WT 9-7 cells were treated with DMSO or 1 nM PAC and 10 nM ATRA for 48 h. (a) To observe the morphology of the nucleus, the cells were stained with Hoechst 33342. Photographs were taken under a fluorescence microscope (200x, scale bar indicates 100 *μ*m). White arrows indicate fragmented nuclei, and red arrows represent multilobe nuclei. (b, c) To examine cellular apoptosis, cells were treated with 1 nM PAC and 10 nM ATRA for different time periods (upper panel for 48 h, middle panel for 72 h, and lower panel for 96 h) and then incubated with FITC-Annexin V in a buffer containing PI and analyzed by flow cytometry. ^∗^*p* < 0.05 vs. DMSO, ^#^*p* < 0.05. (d) To determine the apoptosis mechanism, protein expression was examined by western blotting. WT 9-7 cells were treated with DMSO or 1 nM PAC plus 10 nM ATRA for 72 h. Cells were then collected and subjected to western blotting to detect p53, cyclin B1, caspase-3, caspase-8, and caspase-9 expression. (e) Densitometric analysis of protein bands was performed by ImageJ software. The band density of each protein was normalized against that of actin. ^∗^*p* < 0.05.

As PAC [8] or ATRA alone [22, 23] can induce cell cycle arrest and apoptosis, we examined cell cycle arrest (Figure 2) and apoptosis (Figure 3) following treatment with PAC+ATRA. After 48 h of treatment, cell cycle progression was analyzed by flow cytometry. The percentage of the cells distributed in the G2/M phase was higher in the PAC+ATRA group than in the control group, while that of the cells in the G0/G1 phase was lower in the combination treatment group than in the control group (Figure 2). This result suggests that the treatment with PAC+ATRA leads to cell cycle arrest at the G2/M phase, probably owing to the reduction in cell proliferation. We investigated the apoptosis rate and found that the cells exhibited multilobe and fragmented nuclei, a typical indication of apoptotic cells after 48 h of combination treatment. The DMSO control cells, on the other hand, presented normal morphology, uniform size, and round shape (Figure 3(a)). The percentages of apoptotic cells significantly increased after 72 and 96 h of PAC+ATRA treatment (Figures 3(b) and 3(c)). Next, we examined the expression of several proteins associated with cell cycle arrest and apoptosis, including p53, cyclin B1, and caspases, by western blotting. The expression of p53, which plays a key role in cell cycle arrest and apoptosis, was upregulated, while the expression of cyclin B1 was decreased in the combination treatment group (Figures 3(d) and 3(e)). The expression of cleaved caspase-8 was markedly induced without any changes in caspase-9 and caspase-3 (Figures 3(d) and 3(e)).

### 3.3. Combination Treatment with PAC and ATRA Increases Intracellular Ca^2+^ Levels

**Figure 4:**
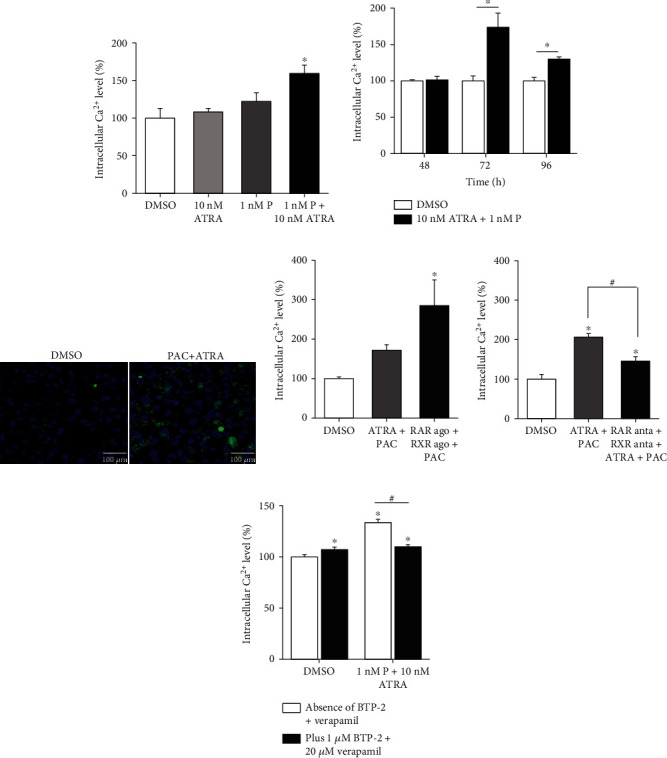
Combination treatment with PAC and ATRA increases intracellular Ca^2+^ level in a RAR/RXR-dependent manner. (a) WT 9-7 cells were treated with different agents for 72 h. (b) WT 9-7 cells were treated with DMSO or 10 nM ATRA plus 1 nM PAC for different time periods as indicated. The cells were then collected, and the intracellular Ca^2+^ levels were examined by flow cytometry. (c) WT 9-7 cells were treated with DMSO or 10 nM ATRA plus 1 nM PAC for 72 h, and the intracellular Ca^2+^ level in ADPKD cells was visualized by fluorescence microscopy (200x, scale bar indicates 100 *μ*m). (d) WT 9-7 cells were treated with 1 nM PAC and 100 nM RAR or RXR agonist for 72 h. (e) Cells were then incubated with 1 nM PAC and 10 nM ATRA in the presence of 100 nM RAR or RXR antagonist for 72 h. Cells were collected, and the intracellular Ca^2+^ level was measured by flow cytometry. ^∗^*p* < 0.05 vs. DMSO, ^#^*p* < 0.05. (f) WT 9-7 cells were treated with DMSO or 1 nM PAC plus 10 nM ATRA in the presence of calcium channel blockers 1 *μ*M BTP-2 and 20 *μ*M verapamil for 72 h. Cells were then collected, and the intracellular Ca^2+^ level was measured by flow cytometry. ^∗^*p* < 0.05 vs. DMSO, ^∗∗^*p* < 0.01, ^#^*p* < 0.05.

As the defects in PC-1/2 function cause reduction in intracellular Ca^2+^ levels in ADPKD cells [3], we examined the intracellular Ca^2+^ level by flow cytometry. Intracellular Ca^2+^ levels increased after PAC+ATRA treatment (Figures 4(a) and 4(b)), as confirmed by fluorescence microscopy (Figure 4(c)). Furthermore, the treatment of the cells with RAR/RXR agonists, instead of ATRA, led to an increase in intracellular Ca^2+^ levels (Figure 4(d)), and that with the RAR/RXR antagonist reversed the PAC+ATRA-induced increase in intracellular Ca^2+^ level (Figure 4(e)). Thus, the increase in intracellular Ca^2+^ levels is assumed to be associated with the canonical RAR/RXR pathway. To determine whether the plasma membrane Ca^2+^ channels are involved in PAC+ATRA-induced increase in intracellular Ca^2+^ levels, we used two different Ca^2+^ channel inhibitors, namely, verapamil that blocks a voltage-gated Ca^2+^ channel and BTP-2 that blocks a Ca^2+^ release-activated channel. As shown in Figure 4(f), the treatment with both inhibitors reversed the PAC+ATRA-induced increase in cellular Ca^2+^ levels, suggesting that Ca^2+^ uptake through the plasma Ca^2+^ channel is responsible for the PAC+ATRA-induced increase in cellular Ca^2+^ levels.

### 3.4. Combination Treatment with PAC and ATRA Suppresses ERK Signaling

**Figure 5:**
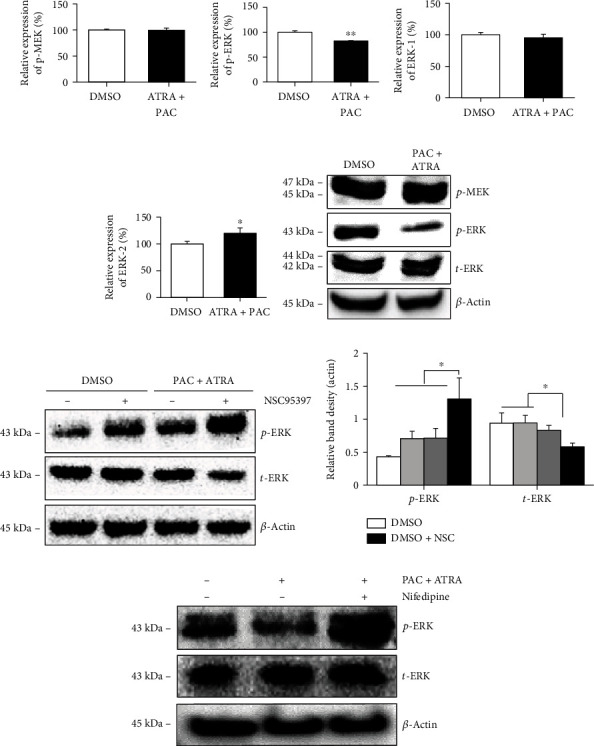
Combination treatment with PAC and ATRA suppresses ERK signaling. Protein expression was examined by flow cytometry analysis and western blotting. WT 9-7 cells were treated with DMSO or 1 nM PAC and 10 nM ATRA for 72 h. Cells were then collected, and the expression of p-MEK (a), p-ERK (b), ERK1 (c), and ERK2 (d) was measured by flow cytometry. (e) Expression levels of these signaling molecules were confirmed by western blotting. (f) WT 9-7 cells were incubated with 20 *μ*M MKP inhibitor (NSC95397) for 6 h and then treated with 1 nM PAC and 10 nM ATRA. After 72 h, the cells were collected and ERK expression was examined by western blotting. (g) Densitometric analysis of protein bands was performed by ImageJ software. The band density of each protein was normalized against that of actin. ^∗^*p* < 0.05. (h) WT 9-7 cells were treated with DMSO or 1 nM PAC plus 10 nM ATRA in the presence of 10 *μ*M nifedipine for 72 h. Cells were then collected, and p-ERK expression was examined by western blotting.

As abnormal renal epithelial cell proliferation in ADPKD is associated with cAMP-induced activation of the B-Raf/MEK/ERK pathway [24], we attempted to examine whether the treatment with PAC+ATRA affects the MEK/ERK pathway and consequently controls ADPKD epithelial cell growth. Flow cytometry and western blot analysis revealed that the protein expression level of p-MEK in PAC+ATRA-treated cells did not differ from that in DMSO-treated cells (Figures 5(a) and 5(e)). However, the expression level of p-ERK decreased in the PAC+ATRA treatment group (Figures 5(b) and 5(e)), and that of ERK was slightly different or unchanged (Figures 5(c)–5(e)). Thus, the antiproliferative effect of PAC+ATRA treatment on ADPKD cells is assumed to be mainly mediated through the suppression of the ERK signaling but not the classical MEK/ERK pathway. As MKP-1 is known to deactivate ERK [25], we examined whether PAC+ATRA-induced suppression of ERK phosphorylation is reversed by treatment with an MKP-1-specific inhibitor NSC 95397 [26]. Western blotting results showed that the decreased expression level of p-ERK in PAC+ATRA-treated cells was restored following treatment with the inhibitor (Figures 5(f) and 5(g)), suggesting that PAC+ATRA-induced ERK inhibition is associated with MKP-1 activity. To gain more insight into the crosstalk between PAC+ATRA-induced intracellular Ca^2+^ and ERK inhibition, we used nifedipine, one of the most common L-type calcium channel blockers, to disrupt the calcium influx; then, the phosphorylation of ERK was examined. Our result showed that the PAC+ATRA-reduced p-ERK level was restored in the presence of nifedipine (Figure 5(h)).

**Figure 6:**
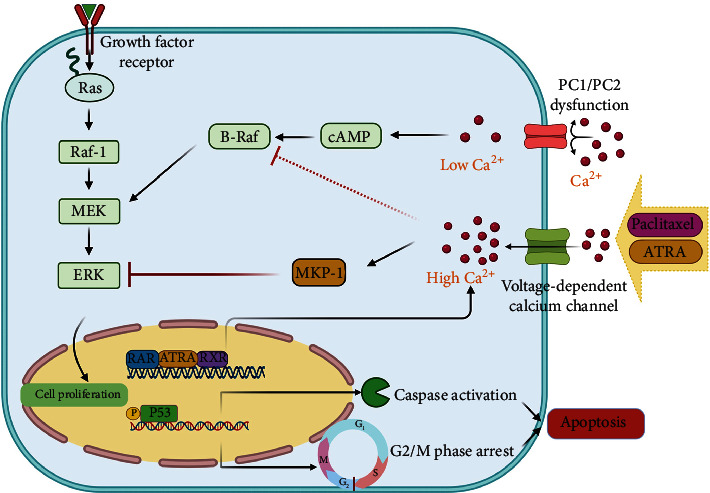
Proposed mechanisms underlying the antiproliferative effect of the combination of PAC and ATRA on ADPKD cells. In ADPKD, the lower intracellular Ca^2+^ level owing to PC1/PC2 dysfunction results in the activation of the mitogenic effect of cAMP-dependent B-raf, thereby triggering cell proliferation through MEK/ERK activation. The stimulation with PAC and ATRA results in the activation of the plasma membrane Ca^2+^ channels, leading to an increase in the intracellular Ca^2+^ levels in ADPKD cells. Enhanced Ca^2+^ levels activate MKP-1, which subsequently inhibits ERK phosphorylation. As a result, these upstream events induce cell cycle arrest and apoptosis, consequently suppressing ADPKD cell proliferation. The dotted line indicates a mechanism proposed by Yamaguchi et al. [24] that increased intracellular Ca^2+^ levels can suppress cAMP-dependent B-Raf activation.

Based on all the results, we propose a schematic diagram of the pathways contributing to the suppression of cell growth in ADPKD following treatment with PAC+ATRA (Figure 6).

## 4. Discussion

Overgrowth of renal tubular epithelial cells is one of the features characterizing ADPKD. Several pathways, such as mTOR [27], Wnt [28], and B-Raf/MEK/ERK pathways [29], are known to be involved in this hyperproliferation of ADPKD epithelial cells. Thus, most antiproliferative therapies for ADPKD to date have been designed to target these pathways. However, the single-drug treatment appears insufficiently effective or safe for ADPKD; therefore, the combination therapies are currently under investigation. Similar to previous studies [11, 30], treatment with high-dose PAC alone (≥100 nM) significantly reduced cell proliferation, whereas that with 1 nM PAC alone exerts only mild effect. However, combination treatment of 1 nM PAC and 10 nM ATRA could reduce cell proliferation more efficiently than that of high-dose PAC alone. Therefore, the combination treatment of these drugs may be useful to spare some of the undesirable side effects of high-dose PAC treatment. In addition, our results revealed that the HREC remains unaffected, while the ADPKD cell proliferation is significantly reduced by combination treatment with PAC+ATRA. This could be explained by the fact that normal cells maintain calcium homeostasis by regulating the uptake of calcium [31], thus preventing calcium level overload, which is associated with apoptosis [32].

Herein, we demonstrate, for the first time, the synergistic antiproliferative effect of PAC+ATRA on ADPKD cells. These effects may be mediated by several mechanisms as follows: (1) cell cycle arrest at the G2/M phase, (2) p53- and caspase-8-mediated apoptosis, and (3) ERK inhibition possibly through MKP-1 activation.

The antiproliferative effect of 1 nM PAC and 10 nM ATRA treatment on ADPKD cells was comparable to that of 10 or 100 nM PAC treatment alone, indicating that ATRA could reduce the concentration of PAC by 10 to 100 times to achieve similar antiproliferative effects. This antiproliferative effect may be mediated by PAC+ATRA-induced cell cycle arrest at the G2/M phase because PAC is known to be a mitotic inhibitor and may interfere with the formation of mitotic spindles, thereby inducing cell cycle arrest at the G2/M phase [7]. A previous study has shown that PAC exerts synergistic effects with ATRA by inducing mitotic arrest of cancer cells in the G2/M phase [33]. Our western blot result revealed that PAC+ATRA treatment increases expression of nuclear p53, while it decreased expression of cyclin B1. The mechanism by which p53 controls the G2/M checkpoint involves inhibition of the cyclin-dependent kinase Cdc2 and cyclin B1 that are essential for entry into mitosis [34]. Thus, we suggest that PAC+ATRA-induced G2/M cell cycle arrest in ADPKD cells is associated with an increase in nuclear p53 levels and a decrease in cyclin B1 levels.

In the present study, PAC+ATRA treatment increased the apoptosis of ADPKD cells and upregulated the expression of p53 and the active form of caspase-8 but not caspase-9. The treatment with PAC+ATRA induced cell death more efficiently than the treatment with either drug alone. ATRA is known to downregulate the expression of the antiapoptotic molecule B cell lymphoma-2 (Bcl-2) [35], thus subjecting cells to apoptosis [36, 37]. PAC is also able to induce apoptosis through the inactivation of Bcl-2 [12]. Similar to our result, the combination treatment with PAC and ATRA was shown to promote the apoptosis of U87MG glioma cells through the inactivation of Bcl-2 and upregulation of activated caspase-8 expression [21]. Considering that there exists a crosstalk between p53 and caspase activity during apoptosis [38, 39], our results suggest that PAC+ATRA-induced cell death is mainly attributed to the p53-associated death receptor pathway of caspase-8.

PAC+ATRA treatment also increased the intracellular Ca^2+^ levels that were associated with the RAR/RXR pathway. The decrease in intracellular Ca^2+^ levels, as a result of genetic mutations in PKD, is thought to activate the calcium-inhibitable adenylyl cyclase, leading to an increase in cytosolic cAMP levels [40]. The increased cAMP levels can activate the B-Raf/MEK/ERK pathway and stimulate the proliferation of ADPKD cells [24]. Conversely, the addition of Ca^2+^ could completely reverse the aberrant mitogenic response to cAMP in ADPKD cells by blocking cAMP-dependent B-Raf and ERK activation [24]. Thus, we believe that the PAC+ATRA-induced restoration of intracellular Ca^2+^ levels can suppress the intracellular signaling through the B-Raf/MEK/ERK pathway. Indeed, our results have demonstrated that PAC+ATRA treatment reduces the phosphorylation of ERK without affecting the phosphorylation of MEK, which is an upstream signaling molecule in the MAPK pathway. Therefore, PAC+ATRA treatment may directly reduce ERK phosphorylation without affecting the upstream kinase in the MAPK pathway. Taken together with the result obtained from the experiment using the MKP-1 inhibitor, we suggest that PAC+ATRA treatment inhibits ERK activation possibly through MKP-1 activation without affecting MEK activity. However, previous studies have demonstrated that PAC inhibits cell proliferation through inhibition of the p38/JNK [41] or PI3K/AKT signaling pathway [42]. In addition, ATRA-induced cell growth inhibition is mediated through the inhibitory action on JNK that plays a critical role in the regulation of cell growth by increasing MKP-1 activity through a retinoid receptor-dependent mechanism [43, 44]. Thus, the possible involvement of signaling pathways other than the ERK MAPK pathway cannot be excluded.

Considering these consecutive molecular signaling processes in ADPKD cells, the PAC+ATRA-induced restoration of cytosolic Ca^2+^ levels is the most upstream event and probably the most important mechanism underlying the suppression of cAMP-induced ADPKD epithelial cell proliferation. The increase in cytosolic Ca^2+^ levels in the presence of either ATRA or PAC alone has been reported, but the exact molecular mechanism for this is yet unclear. In the present study, we have demonstrated that the plasma membrane Ca^2+^ channels are associated with the PAC+ATRA-induced increase in intracellular Ca^2+^ levels. However, the mechanism for this effect is currently unknown. Thus, it is necessary to search for membrane Ca^2+^ channels responsible for PAC+ATRA-induced restoration of intracellular Ca^2+^ levels in future study.

Given that Ca^2+^ is necessary and sufficient for the induction [45] and maintenance [46] of MKP-1 expression and MKP-1 is a primary phosphatase for deactivating ERK [47], the PAC+ATRA-induced intracellular Ca^2+^ restoration might be responsible for MKP-1-induced deactivation of ERK. As ADPKD cells can be rescued by increasing intracellular Ca^2+^ levels [24] and cAMP-dependent activation of the B-Raf/MEK/ERK pathway is thought to be the key event underlying the aberrant cell proliferation in PKD [48], the PAC+ATRA-induced intracellular Ca^2+^ restoration and subsequent ERK inhibition can be considered the potential therapeutic mechanism for ADPKD.

In conclusion, the current investigation demonstrates that the combination of ATRA and PAC synergistically suppressed ADPKD cell proliferation through different molecular mechanisms. Further in vitro cyst formation and in vivo studies in the murine PKD model would reveal the potential application of this combination treatment regimen for ADPKD.

## Data Availability

The data used to support the findings of this study are available from the corresponding author upon request.

## Abbreviations

ADPKD: Autosomal dominant polycystic kidney disease
ATRA: All-trans retinoic acid
PAC: Paclitaxel
RAR: Retinoic acid receptor
RXR: Retinoid X receptor.

## References

[1] Torres V. E., Harris P. C., Pirson Y. (2007). Autosomal dominant polycystic kidney disease. *Lancet*.

[2] Dell K. M. (2011). The spectrum of polycystic kidney disease in children. *Advances in Chronic Kidney Disease*.

[3] Martínez V. (2014). Autosomal dominant polycystic kidney disease: review and management update. *European Medical Journal Nephrology*.

[4] Harris P. C., Torres E. (2014). Genetic mechanisms and signaling pathways in autosomal dominant polycystic kidney disease. *Journal of Clinical Investigation*.

[5] Mao Z., Chong J., Ong A. C. (2016). Autosomal dominant polycystic kidney disease: recent advances in clinical management. *F1000Research*.

[6] Woo D. D. L., Miao S. Y. P., Pelayo J. C., Woolf A. S. (1994). Taxol inhibits progression of congenital polycystic kidney disease. *Nature*.

[7] Horwitz S. B. (1992). Mechanism of action of taxol. *Trends in Pharmacological Sciences*.

[8] Liao P. C., Tan S. K., Lieu C. H., Jung H. K. (2008). Involvement of endoplasmic reticulum in paclitaxel-induced apoptosis. *Journal of Cellular Biochemistry*.

[9] Wang T. H., Wang H. S., Soong Y. K. (2000). Paclitaxel-induced cell death where the cell cycle and apoptosis come together. *Cancer*.

[10] Kucukzeybek Y., Gul M. K., Cengiz E. (2008). Enhancement of docetaxel-induced cytotoxicity and apoptosis by all-trans retinoic acid (ATRA) through downregulation of survivin (BIRC5), MCL-1 and LTbeta-R in hormone- and drug resistant prostate cancer cell line, DU-145. *Journal of Experimental & Clinical Cancer Research*.

[11] Park S. J., Wu C. H., Gordon J. D., Zhong X., Emami A., Safa A. R. (2004). Taxol Induces Caspase-10-dependent Apoptosis∗. *Journal of Biological Chemistry*.

[12] Haldar S., Jena N., Croce C. M. (1995). Inactivation of Bcl-2 by phosphorylation. *Proceedings of the National Academy of Sciences*.

[13] Clegg A., Scott D. A., Hewitson P., Sidhu M., Waugh N. (2002). Clinical and cost effectiveness of paclitaxel, docetaxel, gemcitabine, and vinorelbine in non-small cell lung cancer: a systematic review. *Thorax*.

[14] Kurie J. M., Hong W. K. (1999). Retinoids as antitumor agents: a new age of biological therapy. *The Cancer Journal from Scientific American*.

[15] Lotan R. (1996). Retinoids in cancer chemoprevention. *The FASEB Journal*.

[16] Smith M., Parkinson D., Cheson B. D., Friedman M. A. (1992). Retinoids in cancer therapy. *Journal of Clinical Oncology*.

[17] Blutt S. E., Allegretto E. A., Pike J. W., Weigel N. L. (1997). 1,25-Dihydroxyvitamin D3 and 9-cis-retinoic acid act synergistically to inhibit the growth of LNCaP prostate cells and cause accumulation of cells in G1. *Endocrinology*.

[18] Caliaro M., Vitaux P., Lafon C. (1997). Multifactorial mechanism for the potentiation of cisplatin (CDDP) cytotoxicity by all- _*trans*_ retinoic acid (ATRA) in human ovarian carcinoma cell lines. *British Journal of Cancer*.

[19] Koshiuka K., Elstner E., Williamson E., Said J. W., Tada Y., Koeffler H. P. (2000). Novel therapeutic approach: organic arsenical (melarsoprol) alone or with _*all*-*trans*_ -retinoic acid markedly inhibit growth of human breast and prostate cancer cells _*in vitro*_ and _*in vivo*_. *British Journal of Cancer*.

[20] Nehmé A., Varadarajan P., Sellakumar G. (2001). Modulation of docetaxel-induced apoptosis and cell cycle arrest by all- _trans_ retinoic acid in prostate cancer cells. *British Journal of Cancer*.

[21] Karmakar S., Banik N. L., Ray S. K. (2008). Combination of all-trans retinoic acid and paclitaxel-induced differentiation and apoptosis in human glioblastoma U87MG xenografts in nude mice. *Cancer*.

[22] Su B., Chen X., Zhong C., Guo N., He J., Fan Y. (2012). All-trans retinoic acid inhibits mesangial cell proliferation by up-regulating p21Waf1/Cip1 and p27Kip1 and down-regulating Skp2. *Journal of Nephrology*.

[23] Witcher M., Ross D. T., Rousseau C., Deluca L., Miller WH Jr (2003). Synergy between all-trans retinoic acid and tumor necrosis factor pathways in acute leukemia cells. *Blood*.

[24] Yamaguchi T., Hempson S. J., Reif G. A., Hedge A. M., Wallace D. P. (2006). Calcium restores a normal proliferation phenotype in human polycystic kidney disease epithelial cells. *Journal of the American Society of Nephrology*.

[25] Wancket L. M., Frazier W. J., Liu Y. (2012). Mitogen-activated protein kinase phosphatase (MKP)-1 in immunology, physiology, and disease. *Life Sciences*.

[26] Vogt A., McDonald P. R., Tamewitz A. (2008). A cell-active inhibitor of mitogen-activated protein kinase phosphatases restores paclitaxel-induced apoptosis in dexamethasone-protected cancer cells. *Molecular Cancer Therapeutics*.

[27] Conduit S. E., Davies E. M., Ooms L. M. (2020). AKT signaling promotes DNA damage accumulation and proliferation in polycystic kidney disease. *Human Molecular Genetics*.

[28] Benzing T., Simons M., Walz G. (2007). Wnt signaling in polycystic kidney disease. *Journal of the American Society of Nephrology*.

[29] Parker M. I., Nikonova A. S., Sun D., Golemis E. A. (2020). Proliferative signaling by ERBB proteins and RAF/MEK/ERK effectors in polycystic kidney disease. *Cellular Signalling*.

[30] Ren X., Zhao B., Chang H., Xiao M., Wu Y., Liu Y. (2018). Paclitaxel suppresses proliferation and induces apoptosis through regulation of ROS and the AKT/MAPK signaling pathway in canine mammary gland tumor cells. *Molecular Medicine Reports*.

[31] Bronner F. (2001). Extracellular and intracellular regulation of calcium homeostasis. *The Scientific World Journal*.

[32] Orrenius S., Gogvadze V., Zhivotovsky B. (2015). Calcium and mitochondria in the regulation of cell death. *Biochemical and Biophysical Research Communications*.

[33] Vivat-Hannah V., You D., Rizzo C. (2001). Synergistic cytotoxicity exhibited by combination treatment of selective retinoid ligands with taxol (paclitaxel). *Cancer Research*.

[34] Taylor W. R., Stark G. R. (2001). Regulation of the G2/M transition by p53. *Oncogene*.

[35] Xia L., Wurmbach E., Waxman S., Jing Y. (2006). Upregulation of Bfl-1/A1 in leukemia cells undergoing differentiation by all- _trans_ retinoic acid treatment attenuates chemotherapeutic agent-induced apoptosis. *Leukemia*.

[36] Warner H. R. (1997). Aging and regulation of apoptosis. *Current Topics in Cellular Regulation*.

[37] Choi Y. H. (2006). Apoptosis of U937 human leukemic cells by sodium butyrate is associated with inhibition of telomerase activity. *International Journal of Oncology*.

[38] Ozaki T., Nakagawara A. (2011). Role of p53 in cell death and human cancers. *Cancers*.

[39] Schuler M., Green D. (2001). Mechanisms of p53-dependent apoptosis. *Biochemical Society Transactions*.

[40] Wang X., Ward C. J., Harris P. C., Torres V. E. (2010). Cyclic nucleotide signaling in polycystic kidney disease. *Kidney International*.

[41] Ruan D., Li X., Li A. (2017). Paclitaxel inhibits growth and proliferation of glioblastoma through MMP-9-meidated p38/JNK signaling pathway. *Biomedical Research*.

[42] Li G., Xu D., Sun J., Zhao S., Zheng D. (2020). Paclitaxel inhibits proliferation and invasion and promotes apoptosis of breast cancer cells by blocking activation of the PI3K/AKT signaling pathway. *Advances in Clinical and Experimental Medicine*.

[43] Lee H. Y., Sueoka N., Hong W. K., Mangelsdorf D. J., Claret F. X., Kurie J. M. (1999). All-trans-retinoic acid inhibits Jun N-terminal kinase by increasing dual-specificity phosphatase activity. *Molecular and Cellular Biology*.

[44] Palm-Leis A., Singh U. S., Herbelin B. S., Olsovsky G. D., Baker K. M., Pan J. (2004). Mitogen-activated Protein Kinases and Mitogen-activated Protein Kinase Phosphatases Mediate the Inhibitory Effects of All- _trans_ Retinoic Acid on the Hypertrophic Growth of Cardiomyocytes. *Journal of Biological Chemistry*.

[45] Scimeca J. C., Servant M. J., Dyer J. O., Meloche S. (1997). Essential role of calcium in the regulation of MAP kinase phosphatase-1 expression. *Oncogene*.

[46] Ciccarelli M., Rusciano M. R., Sorriento D. (2014). CaMKII protects MKP-1 from proteasome degradation in endothelial cells. *Cellular Signalling*.

[47] Kidger A. M., Keyse S. M. (2016). The regulation of oncogenic Ras/ERK signalling by dual-specificity mitogen activated protein kinase phosphatases (MKPs). *Seminars in Cell and Developmental Biology*.

[48] Calvet J. P. (2006). MEK inhibition holds promise for polycystic kidney disease. *Journal of the American Society of Nephrology*.

